# Combining QTL-seq and linkage mapping to uncover the genetic basis of single vs. paired spikelets in the advanced populations of two-ranked maize×teosinte

**DOI:** 10.1186/s12870-021-03353-3

**Published:** 2021-12-04

**Authors:** Zhengjie Chen, Dengguo Tang, Kun Hu, Lei Zhang, Yong Yin, Jixing Ni, Peng Li, Le Wang, Tingzhao Rong, Jian Liu

**Affiliations:** 1 Maize Research Institute, Sichuan Agricultural University, No.211 Huiming Road, Wenjiang District, Chengdu, 611130 Sichuan China; 2 Industrial Crop Research Institute, Sichuan Academy of Agricultural Science, No.159 Huajin Avanue, Qingbaijiang District, Chengdu, 610300 Sichuan China

**Keywords:** Maize domestication, Single vs. paired spikelets, QTL-seq, Major QTL, Epistasis, Photoperiod

## Abstract

**Background:**

Teosinte ear bears single spikelet, whereas maize ear bears paired spikelets, doubling the number of grains in each cupulate during maize domestication. In the past 20 years, genetic analysis of single vs. paired spikelets (PEDS) has been stagnant. A better understanding of genetic basis of PEDS could help fine mapping of quantitative trait loci (QTL) and cloning of genes.

**Results:**

In this study, the advanced mapping populations (BC_3_F_2_ and BC_4_F_2_) of maize × teosinte were developed by phenotypic recurrent selection. Four genomic regions associated with PEDS were detected using QTL-seq, located on 194.64–299.52 Mb, 0–162.80 Mb, 12.82–97.17 Mb, and 125.06–157.01 Mb of chromosomes 1, 3, 6, and 8, respectively. Five QTL for PEDS were identified in the regions of QTL-seq using traditional QTL mapping. Each QTL explained 1.12–38.05% of the phenotypic variance (PVE); notably, QTL *qPEDS3.1* with the average PVE of 35.29% was identified in all tests. Moreover, 14 epistatic QTL were detected, with the total PVE of 47.57–66.81% in each test. The QTL *qPEDS3.1* overlapped with, or was close to, one locus of 7 epistatic QTL. Near-isogenic lines (NILs) of QTL *qPEDS1.1*, *qPEDS3.1*, *qPEDS6.1*, and *qPEDS8.1* were constructed. All individuals of NIL-*qPEDS6.1*(MT1) and NIL-*qPEDS8.1*(MT1) showed paired spikelets (PEDS = 0), but the flowering time was 7 days shorter in the NIL-*qPEDS8.1*(MT1). The ratio of plants with PEDS > 0 was low (1/18 to 3/18) in the NIL-*qPEDS1.1*(MT1) and NIL-*qPEDS3.1*(MT1), maybe due to the epistatic effect.

**Conclusion:**

Our results suggested that major QTL, minor QTL, epistasis and photoperiod were associated with the variation of PEDS, which help us better understand the genetic basis of PEDS and provide a genetic resource for fine mapping of QTL.

**Supplementary Information:**

The online version contains supplementary material available at 10.1186/s12870-021-03353-3.

## Background

Improvement in grain yield was one of the main goals during maize domestication [1]. Among the many factors that affect the grain yield, grain number per ear is a major determinant. The number of kernels per maize ear had been raised to 200 or more from its closest wild relative teosinte that has 6 to 12 kernels per ear. The single spikelets in teosinte ear were completely transformed into paired spikelets in maize ears, with the two ranks of teosinte ear changed into multiple ranks of maize ears [2]; hence, the number of kernels per row or length of ear in maize was significantly increased compared to that in teosinte [3]. Notably, the transformation of single spikelet into paired spikelets could double the kernel number, which was one of the key steps in maize domestication. Dissecting the genetic architecture of single vs. paired spikelets could improve our understanding about procedures of maize domestication and the genetic mechanism of yield improvement. However, studying of single vs. paired spikelets has not progressed much in the past 20 years, and the genetic basis of single vs. paired spikelets remains poorly known.

Scanning electron microscopy (SEM) analysis of inflorescence development revealed that the inflorescence meristem (IM) transforms into spikelet pair meristems (SPM). Subsequently, each SPM produces two distinct spikelet meristems (SM), one sessile SM and one pedicellate SM [4]. Both sessile and pedicellate SM develop normally in maize ear, resulting in paired spikelets. In contrast, the pedicellate SM is aborted in teosinte ear, leading to single spikelets [5].

Several studies were performed to explore the inheritance of single vs. paired spikelets. As early as 1920, Collins and Kempton showed for the first time that the distributions of single vs. paired spikelets were continuous, deviating from the Mendelian pattern of 3:1 in the maize-teosinte F_2_ population [6, 7]. Later, Langham suggested that single vs. paired spikelets trait was controlled by a single gene [8], but Szabó and Burr inferred that two independent genes on chromosomes 4 and 8 were involved [9]. In contrast, Mangelsdorf [10], Rogers [11] and Doebley et al. [7] indicated that multiple genes plus epistasis governed single vs. paired spikelets. The differences in these results might be due to that different parents were used to develop the segregating populations and different analysis methods were used in these studies. Currently, the number of studies of QTL mapping for single vs. paired spikelets (PEDS) is less than 10. Doebley et al. mapped four QTL related to PEDS in the F_2_ population of maize race chapalote (Sin 2) × *Z. mays Ssp. Mexicana* (Doebley 643), located on chromosomes 1 to 4, with the proportion of phenotypic variance (R^2^) explained ranging from 0.05 to 0.24 [7]. One year later, three additional QTL for PEDS located on chromosomes 5, 6 and 7 were identified in the same dataset by a modified statistical analysis [12]. Doebley et al. constructed another F_2_ population of maize race reventador (Nay 15) × *Z. mays ssp. pamiglumis* (Iltis Cochrane 81), and detected five QTL for PEDS on chromosomes 1, 2, 3, and 10, with the R^2^ of each QTL was 0.07–0.16 [13]. Moreover, Doebley et al. identified four QTL associated with PEDS in the testcross (TC1) population, which were located on chromosomes 1, 3, 9, and 10, explaining 3.0–7.4% of the phenotypic variance [14]. The QTL (QTL-1 L and QTL-3 L) located on chromosomes 1 L and 3 L were repeatedly identified in different studies, explaining an average of 26.85 and 32.70% of the phenotypic variance, respectively [7, 12, 13]. In addition, a significant epistatic interaction between QTL-lL (umc107) and QTL-3 L (umc92) that increased the expression of PEDS was identified in the F_2_ population, and the combined effect of these two QTL was 60% [12, 15]. Nevertheless, the combined value was 7.3% when the maize alleles of these two QTL were introduced into teosinte background, which was much less than that in the F_2_ population, suggesting that a number of epistatic interactions may be involved in the PEDS variation [15].

Traditional QTL mapping typically involves genotyping of dozens to hundreds of individuals in segregating populations, which is time and labor intensive and thus expensive [16, 17]. In order to overcome these disadvantages, the selective genotyping was proposed to enhance the efficiency of QTL mapping through selectively genotyping individuals with extreme phenotypes [18]. Further, the procedures were simplified by bulked sample analysis (BSA), only genotyping four DNA pools, including a paired bulked DNA pools with opposite or extreme phenotypes and a pair of parents [19]. The rapid development of next-generation sequencing (NGS) technologies accelerated the application of BSA in QTL analysis and fine mapping because it overcame the limitations of marker density [20]. Combining with the NGS and BSA, several methods have been developed for identification of QTL/genes for quantitative traits, including SHOREmap [21], X-QTL [22], NGM [23], MutMap [24], QTL-seq [25, 26], and QTG-Seq [27]. In maize, these new methods have been utilized for detecting candidate genomic regions, QTL and genes for both quality and quantitative traits such as plant height [28], maize lethal necrosis [29], resistance to gibberella stalk rot [30], and fertility restoration of maize CMS-C [31].

In this study, a two-ranked maize (SICAU1212) and teosinte (*Z. mays ssp. mexicana*) were used as parents to develop the advanced mapping populations (BC_3_F_2_ and BC_4_F_2_) using phenotypic recurrent selection. Firstly, QTL-seq was performed to rapidly identify candidate genomic regions associated with PEDS in five pairs of high PEDS bulks and the low PEDS bulk in the BC_3_F_2_ populations. Subsequently, traditional QTL mapping was carried out to validate the genomic regions detected by QTL-seq and identify the epistatic interactions for PEDS in the BC_3_F_2_ and BC_4_F_2_ populations. Results from the study will enhance our understanding of the genetic basis of PEDS.

## Results

### Phenotypic diversity in the BC_3_F_2_ populations

**Fig. 1:**
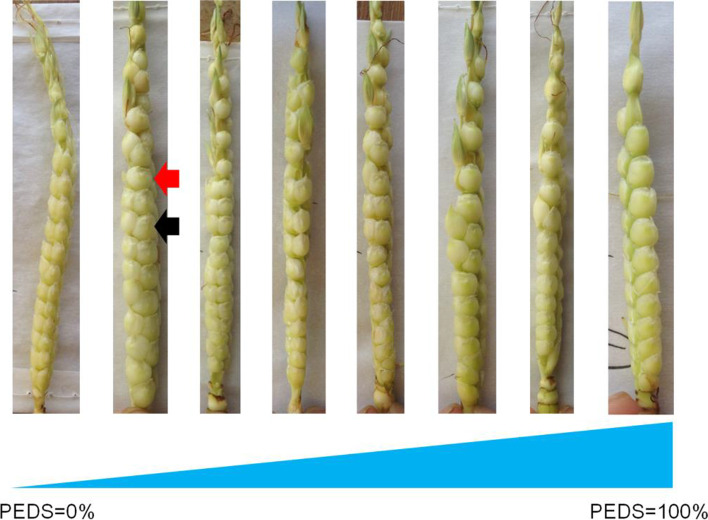
Ears of the plants selected from the BC_3_F_2_ population. The red arrow points to single spikelet, and black arrow points to paired spikelet. PEDS = 0% indicates that all cupules of the ear show paired spikelets, and PEDS = 100% indicates that all cupules of the ear show single spikelet

The ear of maize parent SICAU1212 has four rows (two ranks, two spikelets per cupule), whereas teosinte MT1 has two rows (two ranks, one spikelet per cupule). Therefore, the only difference is paired spikelets in SICAU1212 ear versus single spikelet in MT1 ear. The phenotypic variability of PEDS was high, ranging from PEDS = 0 (same as ear of SICAU1212) to PEDS = 100% (same as ear of MT1) in the BC_3_F_2_ populations (Table 1; Fig. 1). The average PEDS of BC_3_F_2_ populations were about 5.04, 5.19, 4.59, and 0.29% in the 14WJ, 14EEDS, 14JH and 15JH environments. The ranges of PEDS variation were 0 to 100% in the 14WJ, 14EEDS and 14JH environments, but only 0 to 10% in the 15JH environment. The proportions of plants with PEDS > 0% were 1/15.88, 1/14.81, 1/15.25, and 1/17.5 in the four environments (Table 1). Notably, the frequency distributions were continuous variation (except in the 15JH environment) and did not follow normal distribution. Instead, they were dramatically skewed towards the maize phenotype (PEDS = 0%) (Table 1), suggesting that PEDS may be a quantitatively inherited trait.

**Table 1:** Phenotypic variation of PEDS in the BC_3_F_2_ populations in four environments

ENV.^a^	0%	0–10%	10–20%	20–30%	30–40%	40–50%	50–60%	60–70%	70–80%	80–90%	90–100%	Mean (%)	Total	The plants with PEDS> 0%:total
14WJ	4048	1	0	0	2	3	23	35	33	72	103	5.04	4320	1**:**15.88
14EEDS	4683	0	0	3	8	14	25	39	74	88	88	5.19	5022	1**:**14.81
14JH	7466	3	8	23	26	43	65	60	83	93	120	4.59	7990	1**:**15.25
15JH	6336	384	0	0	0	0	0	0	0	0	0	0.29	6720	1**:**17.50

a Env. represents environments.14WJ, 14EEDS, 14JH and 15JH represent Wenjiang 2014, Eerduosi 2014, Jinghong 2014 and Jinghong 2015, respectively

### Whole-genome resequencing, mapping of reads and identification of SNPs

The six bulks and SICAU1212 were sequenced, and the whole-genome resequencing (WGRS) data were generated. A total of 117.17 million paired-end (PE) reads and 34.98 Gb of sequencing data were generated for SICAU1212. The alignment of the PE reads of SICAU1212 to the maize reference genome (B73-RefGen_v4) achieved 80.24% genome coverage and 15.21 × read depth. For the five high PEDS bulks, the number of PE reads ranged from 293.47 to 293.92 million, and the sequencing data for them varied from 44.02 to 44.09 Gb. For the low PEDS bulk, there were 294.18 million PE reads and 44.13 Gb of sequencing data (Table S1). In the case of high PEDS bulks, mapping of reads to the maize reference genome (B73-RefGen_v4) resulted in 81.43 - 85.12% coverage and 19.14 × − 19.17 × read depth. Similarly, in the low PEDS bulk, there was 83.34% coverage and 19.18 × read depth (Table S1). In total, 23,825,847, 24,139,555, 23,686,654, 23,849,835, 23,550,026, and 23,826,344 SNPs were identified for HP1-bulk, HP2-bulk, HP3-bulk, HP4-bulk, HP5-bulk, and LP-bulk, respectively. Among them, 4,224,921, 1,778,418, 4,867,842, 4,964,187, and 2,052,270 polymorphic SNPs were detected between HP1-bulk and LP-bulk, HP2-bulk and LP-bulk, HP3-bulk and LP-bulk, HP4-bulk and LP-bulk, and HP5-bulk and LP-bulk, respectively.

### Candidate genomic regions associated with PEDS

**Fig. 2:**
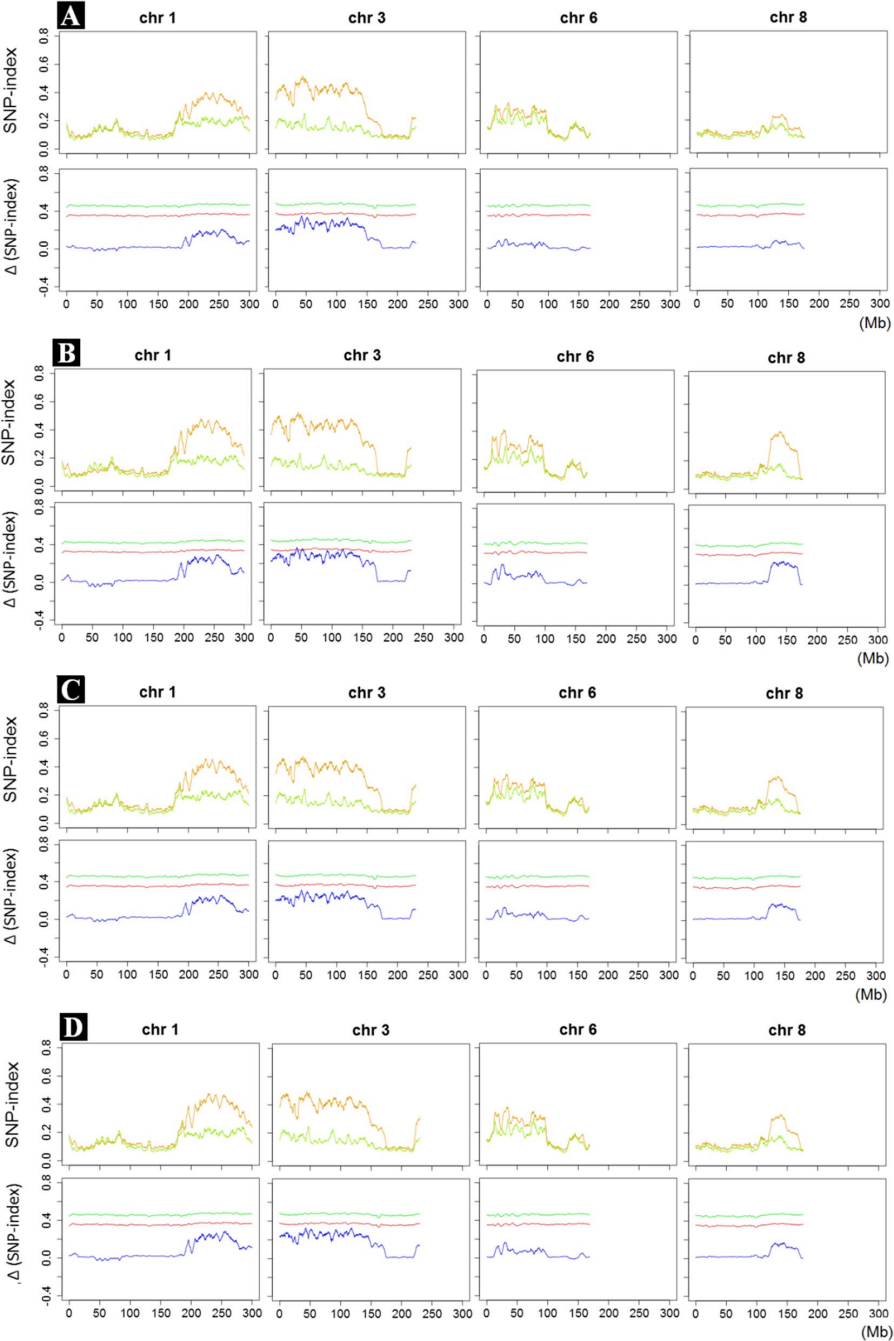
Four candidate genomic regions associated with PEDS identified by QTL-seq. **A** HP1-bulk vs. LP-bulk, **B** HP2-bulk vs. LP-bulk, **C** HP3-bulk vs. LP-bulk, and **D** HP4-bulk vs. LP-bulk. X-axis represents the position of chromosome (Mb) and Y-axis represents the SNP-index or Δ (SNP-index). The light green lines represent the SNP-index of low PEDS bulks, the orange lines represent the SNP-index of high PEDS bulks; the blue lines represent the Δ (SNP-index), the red lines represent the threshold values under the null hypothesis at significant level P < 0.05, and the green lines represent the threshold values under the null hypothesis at significant level *P* < 0.01

To identify candidate genomic regions associated with PEDS, the SNP-index was estimated individually in each bulk and Δ (SNP-index) was calculated, and the corresponding graphs were plotted against the genome positions (Fig. S2). Generally speaking, the SNP-index graphs between high and low bulks would be identical for the genomic regions that are not relevant to the phenotypic difference, while they should exhibit unequal contributions from the two parental genomes if the genomic region harboring QTL contributed to the difference in the phenotype [25, 32]. Based on the graphs of SNP-index of bulks in Fig. 2, the SNP-indices in the regions on chromosome 1 from 194.64 Mb to 299.52 Mb, chromosome 3 from 0 Mb to 162.80 Mb, chromosome 6 from 12.82 Mb to 97.17 Mb, and chromosome 8 from 125.06 Mb to 157.01 Mb were larger in high bulks than the low bulk in the 14EEDS and 14JH environments, but not in the 15JH environment, suggesting that these regions probably contained QTL responsible for PEDS. In contrast, the SNP-indices of the remaining chromosomes were near 0, indicating that these chromosomes were rich in alleles from the maize parent SICAU1212.

Combining the SNP-index and Δ (SNP-index) values, at 95% significance level, three candidate genomic regions (*seqPEDS3.1*, *seqPEDS3.2* and *seqPEDS3.3*) were identified for PEDS on chromosome 3, located on 42.22–44.05 Mb, 52.13–52.58 Mb and 119.6–119.78 Mb in the HP2-bulk and LP-bulk test (Fig. 2B; Table 2). In all three regions, the alleles from teosinte parent MT1 increased PEDS value, whereas those from maize parent SICAU1212 decreased PEDS value. However, no significant candidate genome region was detected in other bulks tests.

**Table 2:** Significant genomic regions for PEDS identified by QTL-seq

Bulks	QTL	Chromosome	Physical interval (Mb)^a^	Δ (SNP-index) range	Significant level
HP2-bulk VS.LP-bulk	*seqPEDS3.1*	3	42.22–44.05	0.35–0.37	*p* < 0.05
	*seqPEDS3.2*	3	52.13–52.58	0.35–0.36	p < 0.05
	*seqPEDS3.3*	3	119.6–119.78	0.35–0.36	p < 0.05

^a^Physical position is based on B73-RefGen_V4 sequence

### Validation of the genomic regions

**Fig. 3:**
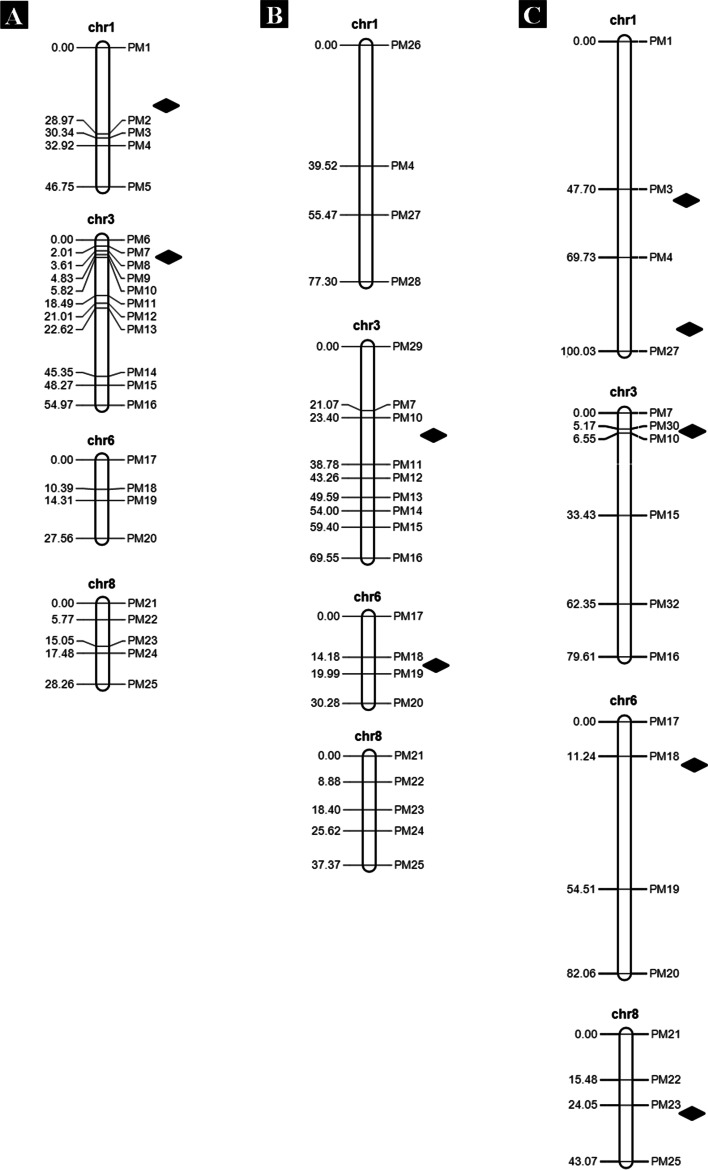
The local genetic linkage maps of BC_3_F_2_ and BC_4_F_2_ populations in three environments. **A** The BC_3_F_2_ population in the 14JH environment, **B** The BC_3_F_2_ population in the 18WJ environment, and **C** The BC_4_F_2_ population in the 15WJ environment. Genetic distances (cM) are shown on the left and markers are indicated on the right. The diamonds represent QTL for PEDS

To validate the genomic regions detected by QTL-seq, the traditional QTL mapping was performed in the BC_3_F_2_ population consisting of 280 and 333 plants from the 14JH and 18WJ environments, and the BC_4_F_2_ population containing 651 plants from the 15WJ environment. In these populations, the frequency distributions did not follow a normal distribution, but were dramatically skewed to maize parent SICAU1212 (Fig. S3). Firstly, 74 markers (1 SSR and 73 InDel) were selected to check polymorphism between parents. Then, the polymorphic markers were utilized to genotype 36 F_2_ plants. Finally, 31 polymorphic and no distorted segregation markers [including 8 markers on chromosome 1, 14 markers on chromosome 3, 4 markers on chromosome 6, and 5 markers on chromosome 8 (Table S2)] were used to genotype the BC_3_F_2_ and BC_4_F_2_ populations. The local genetic linkage maps were constructed for the candidate genomic regions on chromosomes 1, 3, 6, and 8 (Fig. 3).

Using the local genetic linkage maps and the phenotyping data, two and two QTL related to PEDS were identified in the BC_3_F_2_ population in 14WJ and 18WJ environments, and five QTL in the BC_4_F_2_ population in 15WJ environment, located on chromosomes 1, 3, 6 and 8 (Table 3; Fig. 3). The phenotypic variance explained (PVE) of individual QTL ranged from 1.12 to 38.05%. Notably, the QTL *qPEDS3.1* (a major and stably expressed QTL*)* explained 33.91–38.05% of the phenotypic variance identified in all three environments. The additive effect of *qPEDS3.1* was negative, suggesting that the alleles from teosinte parent MT1 increased the value of PEDS. The consistent QTL *qPEDS1.1* and *qPEDS6.1* accounted for 1.12–7.15% of the phenotypic variance in two environments. The QTL *qPEDS1.2* and *qPEDS8.1* with PVE = 1.12–1.82% were only mapped in the BC_4_F_2_ population in 15WJ environment. In summary, the QTL mapping results supported that there were QTL for PEDS in the candidate genomic regions on chromosomes 1, 3, 6 and 8 identified by QTL-seq.

**Table 3:** Significant QTL for PEDS in the BC_3_F_2_ and BC_4_F_2_ populations by traditional QTL mapping

Population	Env.^a^	QTL	Chromosome	Marker interval	Physical interval (Mb)^b^	LOD^c^	PVE (%)^d^	Add	Dom
BC_3_F_2_	14JH	*qPEDS1.1*	1	PM1-PM2	184.63–222.02	5.88	7.15	−0.08	0.07
		*qPEDS3.1*	3	PM7-PM8	10.86–12.56	34.81	38.05	−0.18	− 0.2
	18WJ	*qPEDS3.1*	3	PM10-PM11	14.12–39.65	52.97	33.92	−0.34	− 0.24
		*qPEDS6.1*	6	PM18-PM19	75.43–83.98	3.23	1.12	−0.04	− 0.08
BC_4_F_2_	15WJ	*qPEDS1.1*	1	PM3-PM4	230.13–258.06	5.04	1.25	0.04	−0.05
		*qPEDS1.2*	1	PM4-PM27	258.06–281.12	3.98	1.12	−0.06	− 0.01
		*qPEDS3.1*	3	PM30-PM10	11.89–14.12	31.17	33.91	−0.14	− 0.14
		*qPEDS6.1*	6	PM18-PM19	75.43–83.98	8.2	3.26	0.07	−0.03
		*qPEDS8.1*	8	PM23-PM25	126.24–149.80	4.69	1.82	−0.05	0.03

^a^Env. represents environments.14JH, 18WJ and 15WJ represent Jinghong 2014, Wenjiang 2018 and Wenjiang 2015, respectively

^b^Physical position is based on B73-RefGen_V4 sequence

^c^LOD score

^d^Phenotypic variance explained by each QTL

**Fig. 4:**
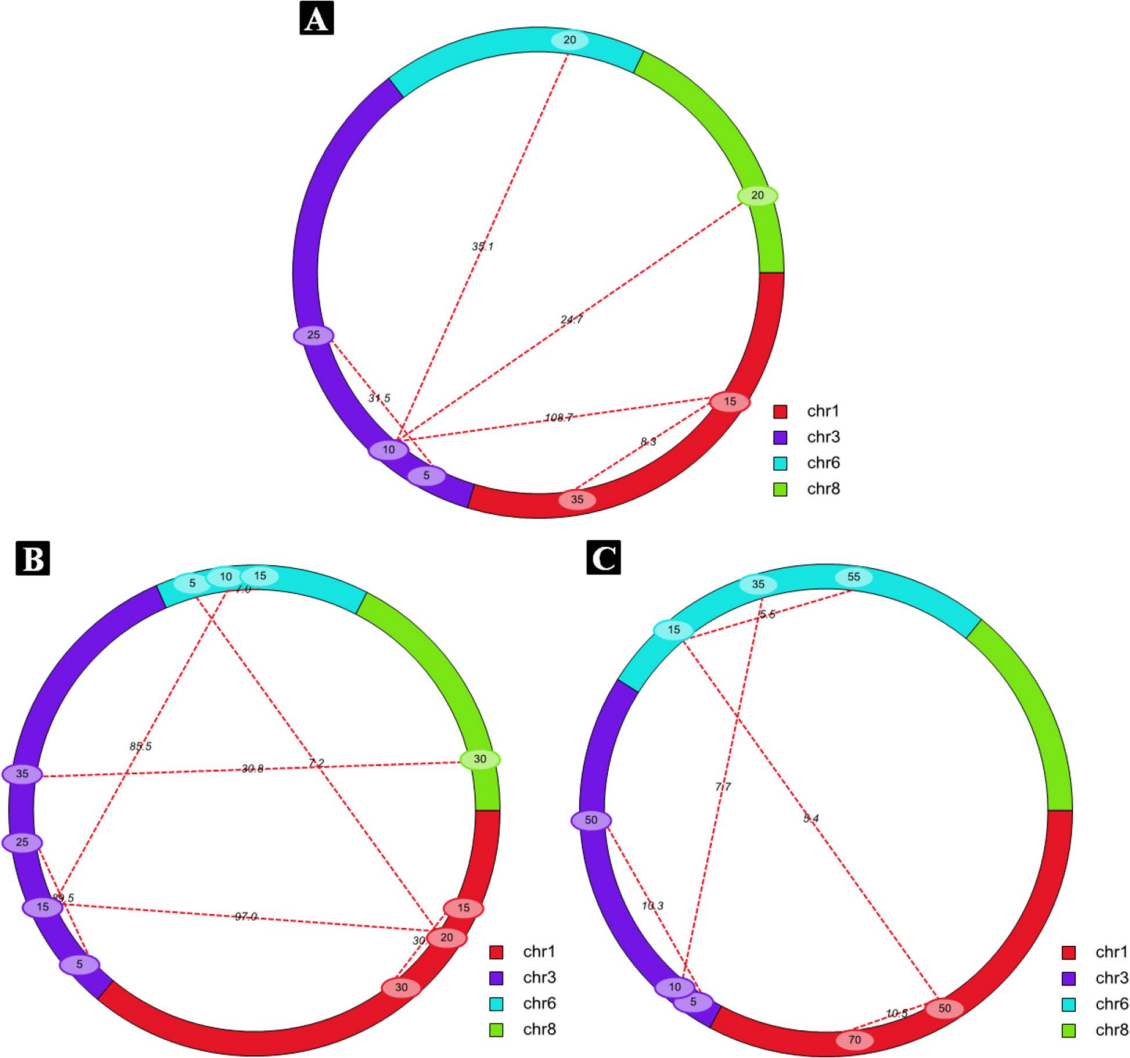
Distribution of epistatic QTL for PEDS identified by ICIM in the BC_3_F_2_ and BC_4_F_2_ populations in three environments. **A** The BC_3_F_2_ population in the 14JH environment, **B** The BC_3_F_2_ population in the 18WJ environment, and **C** The BC_4_F_2_ population in the 15WJ environment. The red long dashed lines attached to the two loci involved in epistatic interaction for PEDS, the numerical values in the colorized circle represent the genetic position on the linkage group, and the numbers on the dashed lines show the LOD values of the corresponding epistatic interactions

Five, seven and five pairs of epistatic QTL were detected in the BC_3_F_2_ population in the 14WJ and 18WJ environments, and the BC_4_F_2_ population in the 15WJ environment, respectively (Table 4; Fig. 4). The PVE of individual epistatic QTL ranged from 3.85 to 21.31%; the total PVE of all the epistatic QTL varied from 45.81 to 66.81%, being larger than those of all the QTL mentioned above in each environment. However, there were two kinds of epistatic QTL [33]. One was that additive by additive effect was positive, increasing the expression of PEDS and explaining 56.85, 34.57 and 18.71% of phenotypic variation in each test, whereas the other was negative, decreasing the expression of PEDS and explaining 9.96, 11.23 and 28.85% of phenotypic variation.

**Table 4:** Epistatic QTL for PEDS identified in the BC_3_F_2_ and BC_4_F_2_ populations in three environments

Population	Env.^a^	epistatic QTL	Chromosome1	Position1 (cM)^b^	Marker interval1	Chromosome2	Position2 (cM)	Marker interval 2	LOD^c^	PVE (%)^d^	AA^e^
BC_3_F_2_	14JH	*EPqpeds-1*	1	15	PM1-PM2	1	35	PM4-PM5	8.26	9.96	−0.14
		*EPqpeds-2*	1	15	PM1-PM2	3	10	PM10-PM11	108.70	16.45	0.21
		*EPqpeds-3*	3	5	PM9-PM10	3	25	PM13-PM14	31.53	12.25	0.00
		*EPqpeds-4*	3	10	PM10-PM11	6	20	PM19-PM20	35.15	14.16	0.20
		*EPqpeds-5*	3	10	PM10-PM11	8	20	PM24-PM25	24.67	13.98	0.17
	18WJ	*EPqpeds-6*	1	15	PM26-PM4	1	30	PM26-PM4	30.39	6.05	0.09
		*EPqpeds-2*	1	20	PM26-PM4	3	15	PM29-PM7	97.00	7.15	0.05
		*EPqpeds-7*	3	5	PM29-PM7	3	25	PM10-PM11	89.50	9.45	0.15
		*EPqpeds-8*	1	20	PM26-PM4	6	5	PM17-PM18	7.22	4.62	0.07
		*EPqpeds-9*	3	15	PM29-PM7	6	10	PM17-PM18	85.49	7.30	0.06
		*EPqpeds-10*	6	10	PM17-PM18	6	15	PM18-PM19	6.98	3.85	−0.10
		*EPqpeds-5*	3	35	PM10-PM11	8	30	PM24-PM25	30.83	7.38	−0.20
BC_4_F_2_	15WJ	*EPqpeds-1*	1	50	PM3-PM4	1	70	PM4-PM27	10.54	3.87	0.08
		*EPqpeds-11*	3	5	PM7-PM30	3	50	PM15-PM32	10.30	10.53	0.10
		*EPqpeds-12*	1	50	PM3-PM4	6	15	PM18-PM19	5.36	7.54	−0.10
		*EPqpeds-13*	3	10	PM10-PM15	6	35	PM18-PM19	7.74	21.31	−0.01
		*EPqpeds-14*	6	15	PM18-PM19	6	55	PM19-PM20	5.47	4.32	0.11

^a^Env. represents environments.14JH, 18WJ and 15WJ represent Jinghong 2014, Wenjiang 2018 and Wenjiang 2015, respectively

^b^The numbers represent the genetic position on the genetic linkage map

^c^LOD score

^d^Phenotypic variation explained by epistatic QTL

^e^Additive by additive effect of QTL at the two scanning positions. The positive and negative values indicate the epistatic QTL increased and decreased the phenotypic value, respectively

### Investigation of PEDS in the near-isogenic lines (NILs)

For each QTL related to PEDS identified by the traditional QTL mapping, NILs were developed through selecting the plants with the MT1 alleles in the target QTL regions and the SICAU1212 alleles in other QTL regions in the BC_4_F_2_ population mentioned above, designated as NIL-*qPEDS1.1*(MT1), NIL-*qPEDS3.1*(MT1), NIL-*qPEDS6.1*(MT1), and NIL-*qPEDS8.1*(MT1). Two families of each NIL with 16–21 plants were grown at Jinghong city, Yunnan province, in September 2015, and the parent SICAU1212 was used as CK. In the NIL-*qPEDS1.1*(MT1), two out of 16 plants in one family showed PEDS > 0, with the average PEDS of 6.91%, but the PEDS of all 18 plants in another family were 0 (Table 5). In the NIL-*qPEDS3.1*(MT1), three and one plants showed PEDS > 0 in the two families with 18 plants, with the average PEDS of 31.43 and 32.14%. However, the PEDS of all plants in the NIL-*qPEDS6.1*(MT1) and NIL-*qPEDS8.1*(MT1) were 0. Notably, the flowering times of plants of NIL-*qPEDS8.1*(MT1) were ~ 7 days earlier compared with SICAU1212. These results indicated that the ratio of plant with PEDS > 0 was low (1/18–3/18) or 0 in each NIL, which was not conducive to fine mapping of QTL.

**Table 5:** Investigation of PEDS in the NILs of QTL

NILs	Genotypes of the NILs^a^				Number of plants	Number of plants with PEDS > 0	PEDS (%)^b^
	*qPEDS1.1*	*qPEDS3.1*	*qPEDS6.1*	*qPEDS8.1*			
NIL-*qPEDS1.1*–1	MT1	SICAU1212	SICAU1212	SICAU1212	16	2	6.91
NIL-*qPEDS1.1*–2	MT1	SICAU1212	SICAU1212	SICAU1212	18	0	0
NIL-*qPEDS3.1*–1	SICAU1212	MT1	SICAU1212	SICAU1212	18	3	31.43
NIL-*qPEDS3.1*–2	SICAU1212	MT1	SICAU1212	SICAU1212	18	1	32.14
NIL-*qPEDS6.1*–1	SICAU1212	SICAU1212	MT1	SICAU1212	16	0	0
NIL-*qPEDS6.1*–2	SICAU1212	SICAU1212	MT1	SICAU1212	17	0	0
NIL-*qPEDS8.1*–1	SICAU1212	SICAU1212	SICAU1212	MT1	16	0	0
NIL-*qPEDS8.1*–2	SICAU1212	SICAU1212	SICAU1212	MT1	21	0	0

^a^MT1 represent the alleles come from the teosinte parent MT1 and SICAU1212 represent the alleles come from the maize parent SICAU1212

^b^The value is the average of PEDS of plants with PEDS > 0

## Discussion

### The two-ranked maize parent SICAU1212 was helpful for accurate identification of PEDS

Single vs. paired spikelets (PEDS) is one of the key domesticated traits. The single spikelets of teosinte ears were totally transformed into paired spikelets of maize ears during maize domestication. However, little is known about the genetic basis of this transformation of single into paired spikelets. Understanding the genetic basis of PEDS is helpful for the map-based gene cloning. In the present study, a primitive four rows (two ranks) waxy maize inbred line (SICAU1212) was used as a female parent to develop the advanced mapping populations (BC_3_F_2_ and BC_4_F_2_). SICAU1212 is similar to the primitive maize of 6000 years ago in phenotypic traits, including small plant, multiple ears, rachis from the ears, 4-row ears and tens of grains [34, 35]. Notably, both the SICAU1212 and teosinte MT1 ears were two ranks, and the ears of all plants in the segregating population of SICAU1212 × MT1 showed only two ranks, thus avoiding a possible confusion about the importance of the single spikelet vs. a decrease in the rank number in multi-rank ears. Therefore, the phenotype of PEDS was investigated more accurately, especially at the silking stage, which was essential for the QTL mapping.

### Comparison of QTL for PEDS in this study with those in previous studies

Four candidate genomic regions associated with PEDS located on chromosomes 1, 3, 6, and 8 were identified using QTL-seq, followed by detecting the QTL for PEDS in each candidate genomic region by traditional QTL mapping. The QTL *qPEDS1.1* identified in this study overlapped with the QTL flanked by umc11-umc83 and umc37b mapped by Doebley et al. [12, 13], because they were all located at 184–258 Mb of chromosome 1. The QTL (QTL-1 L) accounting for ~ 19.5% of phenotypic variance was identified repeatedly in the previous studies [7, 13, 15], near to the gene *teosinte branched1* (*tb1*) [15, 36], which was included in the QTL *qPEDS1.2*. According to the results of QTL-seq, the region of 0–173 Mb on chromosome 3 was associated with PEDS variation; however, only one QTL *qPEDS3.1* explaining 33.91–38.05% of phenotypic variance was identified in the BC_3_F_2_ and BC_4_F_2_ populations by traditional QTL mapping, possibly near to QTL (umc121-umc92 and umc32) mapped by Doebley et al. [7, 14]. The QTL *qPEDS3.1* may be a novel major and stably expressed QTL, and it is worthy of further study. In addition, QTL (QTL-3 L) was identified repeatedly in the previous studies [12, 13, 15], located at ~ 181 Mb on chromosome 3. However, no QTL for PEDS was identified near this region, probably the InDel markers used in this study did not contain this region, with the farthest InDel marker (PM16) being located at ~ 157 Mb on chromosome 3. It is interesting that the region of 0–173 Mb on chromosome 3 was completely retained in the BC_3_F_2_ population developed by phenotypic recurrent selection for PEDS, inferring that several QTL related to PEDS located in this region may not be identified. Although one QTL (umc85-umc65) for PEDS located on chromosome 6 was mapped [12], we do not know how far away it is from the minor QTL *qPEDS6.1* that explained 1.12–3.26% of phenotypic variance because the physical positions of markers umc85 and umc65 could not be found. The QTL *qPEDS8.1* on chromosome 8 L identified in this study may be close to the locus *pd2* related to PEDS mapped by Ve’ronique and Benjamin [9].

### Epistasis played a vital role in PEDS variation

Five to seven epistatic QTL with the total PVE of 47.57–66.81% were identified in each test, which was more than the effect of main QTL (35.02–47.12%). Deobley at el. (1993, 1995) identified significant epistasis between QTL-1 L (umc107) and QTL-3 L (umc92); the effect of each QTL was much lower in the advanced background population than the F_2_ population, with the combined effect of two QTL decreasing about 10-fold [13, 15]. No significant epistatic QTL between QTL-1 L and QTL-3 L was detected in this study; comparatively, one locus of *EPqpeds-1* was included the region of QTL-1 L, one locus of *EPqpeds-6* was close to QTL-1 L, and one locus of *EPqpeds-11* was near QTL-3 L. Notably, one locus of epistatic QTL *EPqpeds-2*, *EPqpeds-3*, *EPqpeds-4*, *EPqpeds-5*, *EPqpeds-7*, and *EPqpeds-13* overlapped with, or was close to, the major QTL *qPEDS3.1* (Table 5); another locus was located on chromosomes 1, 3, 6, and 8, suggesting that the epistatic interactions between *qPEDS3.1* and other loci were very strong, partly explaining the very low ratio of PEDS > 0 in the NIL-*qPEDS3.1*(MT1) families as well as in the other NILs of QTL. Moreover, 50 pairs of epistatic QTL were identified on 10 chromosomes, explaining 94.40% of the total phenotypic variance in the F_2_ population of SICAU1212 × MT1 (unpublished data). The number and effect of epistatic QTL identified in the BC_3_F_2_ and BC_4_F_2_ populations decreased significantly, inferring that the advanced mapping population developed by phenotypic recurrent selection for PEDS eliminated partly the effect of epistasis. Taken together, the epistasis played a key role in PEDS variation. Unfortunately, the impact of epistasis was neglected in fine mapping or positional cloning of QTL/gene for complex traits such as PEDS [37]. For fine mapping of the major QTL *qPEDS3.1*, the two, three and four QTL will be combined to evaluate the effect; then, the residual heterozygous lines of *qPEDS3.1* will be developed in future research by selecting the heterozygous alleles in the region of *qPEDS3.1* and the homozygous alleles in other regions of QTL [38, 39].

### Photoperiod significantly affect the expression of single spikelet

The photoperiod-related trait is a primary domestication trait in maize. The geographic range of maize has rapidly expanded from tropical to temperate regions due to the loss of photoperiod sensitivity [40, 41]. The effects of photoperiod on tassel branches and the number of anthers were reported by Bechoux et al. [42]. Boden et al. suggested that *Photoperiod-1* (*Ppd-1*) responsible for flowering time has a significant inhibitory effect on paired spikelet formation [43]. In the present study, the expression of PEDS decreased significantly, and no genomic region for PEDS was identified in the 15JH environment using QTL-seq (Fig. S2E). Compared to the other three environments, the day length was 11.20 h in the seedling stage and 11.73 h 1 month later in the 15JH environment, which was shorter than 12.53 h in the 14WJ environment, 14.97 h in the 14EEDS environment, and 12.85 h in the 14JH environment. The QTL *qPEDS8.1* was mapped on chromosome 8, which contained the gene *ZCN8* involved in photoperiod sensitivity of tropical maize [44]. Investigation of NIL-*qPEDS8.1*(MT1) planted at Jinghong city, Yunnan province, in September 2015, revealed that the teosinte allele had a shorter flowering time (by about 7 days) than that of SICAU1212. However, the presence of single spikelet was not confirmed. In summary, the results inferred that photoperiod played an important role in the transformation of single into paired spikelets during maize domestication.

## Conclusion

In the present study, to accurately investigate single and paired spikelets, a two-ranked maize were chosen to cross to teosinte to develop the advanced mapping populations (BC3F2 and BC4F2). Four candidate genomic regions associated with PEDS were identified on chromosomes 1, 3, 6 and 8 using QTL-seq. One major QTL, four minor QTL and 14 epistatic QTL for PEDS were detected in these four genomic regions by linkage map analysis. The expression of single spikelet was very low (PEDS < 10%) in the 15JH environment, maybe due to the shorter day length compared to that in the other environments. The flowering time of plants of NIL-*qPEDS8.1*(MT1) was ~ 7 days shorter than that of “parent SICUAN1212”. Taken together, these findings suggested that major QTL, minor QTL, epistasis, and photoperiod were associated with the transformation of single spikelet in teosinte ears into paired spikelets in maize ears during maize domestication, providing insights into the genetic basis of PEDS and an opportunity to fine map major QTL for PEDS in the future work.

## Materials and methods

### Plant materials and development of the advanced mapping populations

The maize inbred line SICAU1212 and teosinte MT1 (*Z. mays ssp. mexicana*) were used as parental lines to develop the advanced segregating populations for PEDS. SICAU1212 was derived from a waxy maize landrace Silunuo [45, 46] and is owned by our lab; it has two ranks with two spikelets per cupule. MT1 was obtained from the International Maize and Wheat Improvement Center (CIMMYT) (accession number 1 l394); it has two ranks with one spikelet per cupule. A cross was made between SICAU1212 and MT1 to create F_1_ in April 2010; F_1_ was self-pollinated to develop the F_2_ population in October 2010. The plants with PEDS = 100% (exclusively singe spikelet) were selected from the F_2_ population to backcross to SICAU1212 to create BC_1_F_1_ in April 2011; then, BC_1_F_1_ was self-pollinated to develop the BC_1_F_2_ population in October 2011. Again, the plants with PEDS = 100% were chosen from the BC_1_F_2_ to backcross to SICAU1212 to create BC_2_F_1_ in April 2012; BC_2_F_1_ was self-pollinated to develop the BC_2_F_2_ population in October 2012. The procedure mentioned above had been performed repeatedly, until the BC_4_F_2_ population was developed in October 2014 (Fig. S1).

### Field experiments and evaluation of PEDS

To investigate the phenotypic values, the BC_3_F_2_ population was grown in five experimental environments, including 4320 plants at Wenjiang district, Sichuan province, in April 2014 (14WJ, 30°40′N, 104°04′E, altitude 750 m, average high / low temperature of 23 °C/ 14 °C in April and 26 °C / 17 °C in May), 5022 plants at Eerduosi city, Neimenggu autonomous region, in June 2014 (14EEDS, 40.04°N, 110.01°E, altitude 1015 m, average high / low temperature of 25 °C/ 15 °C in June and 26 °C / 17 °C in July); 7990 plants at Jinghong city, Yunnan province, in August 2014 (14JH, 22°01′N, 100°58′E, altitude 551 m, average high / low temperature of 31 °C / 22 °C in August and 32 °C / 22 °C in September); 6720 plants at Jinghong city, Yunnan province, in January 2015 (15JH, average high / low temperature of 25 °C / 11 °C in January and 28 °C / 13 °C in February); and 350 plants at Wenjiang district, Sichuan province, in April 2018 (18WJ, average high / low temperature of 25 °C / 14 °C in April and 27 °C / 17 °C in May). The BC_4_F_2_ population of 700 plants was grown at Wenjiang district, Sichuan province, in April 2015 (15WJ, average high / low temperature of 24 °C / 14 °C in April and 28 °C / 18 °C in May).

The presence (paired spikelets)/absence (single spikelet) of the pedicellate spikelet in each cupule can vary among cupules within a single ear; therefore, PEDS was recorded as percentage of cupules without a pedicellate spikelet [7, 12]. For ease and accuracy, single and paired spikelets were counted on the basal-most secondary lateral inflorescence when the plant had silks visible. The cubic root transformations of phenotype data were carried out to reduce skewness and kurtosis because the PEDS distribution of BC_3_F_2_ populations did not follow a normal distribution [12]. The descriptive statistics of the populations were analyzed using SPSS19.0 software (http://www.spss.com).

### Construction of bulks

Extreme bulks were prepared for PEDS based on precise phenotyping. Six extreme bulks were constructed as follows: 50 plants with PEDS > 90% were selected from the BC_3_F_2_ population in the 14EEDS environment to constitute high PEDS bulk (HP1-bulk). One hundred plants with PEDS > 90% were chosen from the BC_3_F_2_ population in the 14JH environment (numbered Y1 to Y100). Three high PEDS bulks were constructed with 25 common plants (Y1-Y25) plus 25 additional plants (Y26-Y50, Y51-Y75, Y76-Y100). HP2-bulk comprised Y1-Y25 + Y26-Y50, HP3-bulk was Y1-Y25 + Y51-Y75, and HP4-bulk was made up of Y1-Y25+ Y76-Y100. Fifty plants with 0% < PEDS ≤10% were selected from the BC_3_F_2_ population in the 15JH environment to create high PEDS bulk (HP5-bulk). Fifty plants with PEDS = 0% were chosen from the BC_3_F_2_ population in the 14JH environment to construct the low PEDS bulk (LP-bulk).

### Construction of sequencing libraries and Illumina sequencing

Genomic DNAs were extracted from the fresh leaves of individuals from the six bulks mentioned above and parent SICAU1212 by using the CTAB method [47]. DNA quality was evaluated using 1% agarose gel, and DNA concentration was quantified using a NanoDrop®. The equimolar concentrations of DNA from individuals of each bulk were pooled together. To construct a library, two micrograms of DNA from each sample were first sheared using diagenode Bioruptor® NGS (Diogenode, Liege, Belgium) and then were subjected to end repair and adapter ligation. The size selection of libraries was performed using 2% agarose gel to get a target insert size of 500–600 bp. The libraries were purified first and then enriched using the adaptor-compatible PCR primers. The size distribution of amplified DNA libraries was checked on an Agilent Technologies 2100 Bioanalyzer using a High Sensitivity chip. The DNA libraries were sequenced on Illumina HiSeqTM 2000 to generate 150-base pair-end reads [48–50].

### Calculation of SNP-index and Δ (SNP-index)

After generating the sequences of all seven samples, the statistical analysis of the sequencing reads was conducted using the raspberry tool of NGS-QCbox [51]. The maize reference genome (B73-RefGen_v4) was downloaded from MaizeGDB (http://www.maizegdb.org/). The cleaned reads of each sample were aligned to B73-RefGen_v4 genome using the inbuilt BWA aligner [52], and the Coval software was used for post-processing and filtering of the alignment files [53]. The SNP-index as the proportion of short reads harboring SNPs that are different from the reference sequence [25, 54], was calculated for each bulk using the formula [24]: SNP-index (at a position) = (count of alternate base) / (count of reads aligned). To improve accuracy of SNPs, the SNP positions with read depth < 4× or > 32× were filtered out because these SNPs may be false positives caused by sequencing, alignment errors, or genomic repeat sequences [25, 32]. An average of SNP-indices of SNPs located in a given genomic interval was calculated using the sliding window analysis with a 1 Mb window size and 10 kb increment [25, 54]. In this study, SNP-index = 1 if all the short reads came from the parent MT1, and SNP-index =0 if all the short reads came from the parent SICAU1212. Δ (SNP-index) was then calculated by subtracting SNP-index of low bulk from SNP index of high bulk. The SNP-index graphs for high bulks and low bulk, and corresponding Δ (SNP-index) Manhattan graphs were plotted against the position of each sliding window in the reference genome (B73-RefGen_v4). The Δ (SNP-index) value of candidate genomic regions associated with a target trait should be significantly different from 0; therefore, candidate genomic regions for PEDS were identified using the hypothesis proposed by Takagi et al. [25].

### Validation of QTL-seq-derived genomic regions by traditional QTL mapping

The candidate genomic regions for PEDS identified by QTL-seq were validated using traditional QTL analysis in three environments. Briefly, InDel markers were identified by aligning high bulks and low bulk, and the primers were designed based on the methods described in the previous study [55]. The InDel markers located on chromosomes 1, 3, 6, and 8 were selected for polymorphism screening between the parents SICAU1212 and MT1. Then, the polymorphic markers were used in genotyping the F_2_ population with 36 plants derived from a cross between SICAU1212 and MT1 to calculate the allele ratios, excluding the markers with serious segregation distortion as determined by chi-square analysis. Subsequently, the markers that followed the expected Mendelian segregation ratio were utilized for genotyping the BC_3_F_2_ populations in the 14JH and 18WJ environments, and the BC_4_F_2_ population in the 15WJ environment. The genotyping data were used to construct the local genetic linkage map using MAPMAKER/EXP 3.0 [56], and the genetic map distances (cM) were calculated following Kosambi mapping function [57]. Combining the genetic linkage information and phenotyping data, QTL analysis for PEDS was conducted with QTL IciMapping 4.1 (http://www.isbreeding.net/) using the inclusive composite interval mapping of additive (ICIM-ADD) and the two locus epistatic QTL (ICIM-EPI) modules [33].

## Supplementary Information

**Additional file 1: Figure S1.** Diagram of the construction of the advanced mapping population through phenotypic recurrent selection. **Figure S2.** SNP-index graphs of high PEDS bulks and low PEDS bulk, and Δ (SNP-index) graphs in three environments. **Figure S3.** Frequency distribution of BC_3_F_2_ and BC_4_F_2_ populations used for QTL mapping in three environments.

**Additional file 2: Table S1.** Sequencing of maize parent line and bulks, and mapping of sequence reads. **Table S2.** The information on markers used in traditional QTL mapping.

**Additional file 3: Table S3.** Genotypes and phenotypes of the mapping populations using traditional QTL mapping.

## Data Availability

The raw data and materials available on request from the authors.

## Abbreviations

PEDS: Percentage of cupules lacking the pedicellate spikelet
ICIM: Inclusive composite interval mapping
QTL: Quantitative trait locus/loci
SEM: Scanning electron microscopy
IM: Inflorescence meristem
SPM: Spikelet pair meristems
SM: Spikelet meristems
PVE: Phenotypic variation explained
BSA: Bulked sample analysis
NGS: Next-generation sequencing
