# Supplementary material for: Combining QTL-seq and linkage mapping to uncover the genetic basis of single vs. paired spikelets in the advanced populations of two-ranked maize×teosinte
Source: BMC Plant Biol. 2021 Dec 4;21:572. doi: 10.1186/s12870-021-03353-3 (PMC8642974; doi:10.1186/s12870-021-03353-3)
Supplement: Supplementary file 1 — Additional file 1: Figure S1. Diagram of the construction of the advanced mapping population through phenotypic recurrent selection. Figure S2. SNP-index graphs of high PEDS bulks and low PEDS bulk, and Δ (SNP-index) graphs in three environments. Figure S3. Frequency distribution of BC3F2 and BC4F2 populations used for QTL mapping in three environments. [file 12870_2021_3353_MOESM1_ESM.docx]

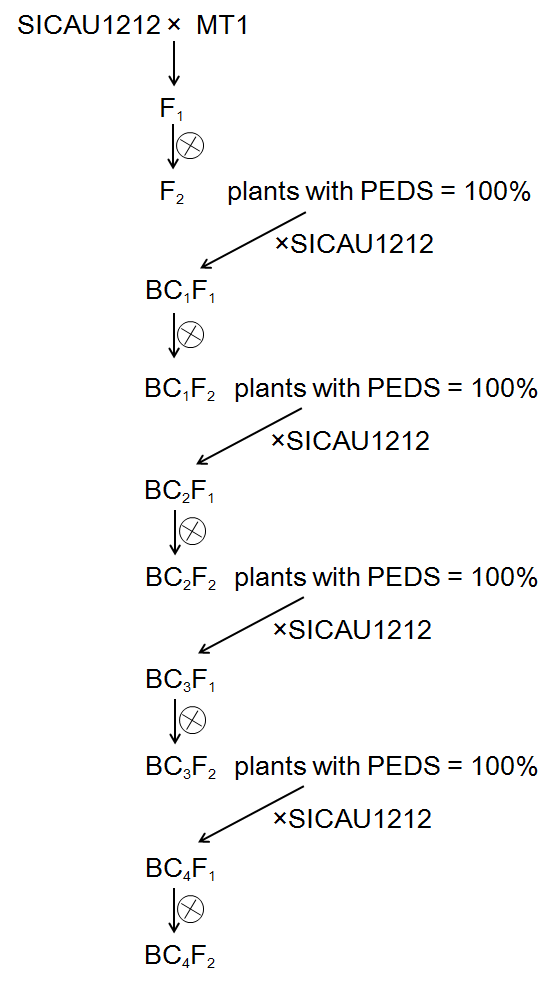


**Figure S1.** Diagram of the construction of the advanced mapping population through phenotypic recurrent selection


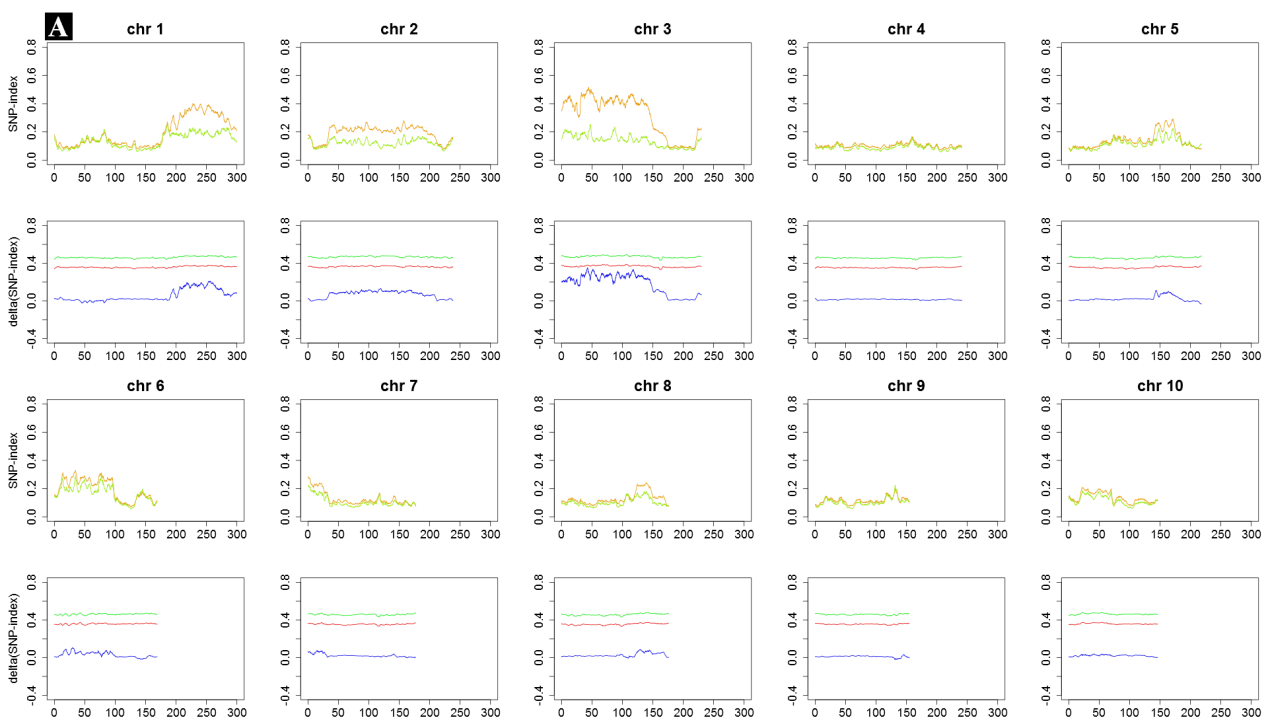

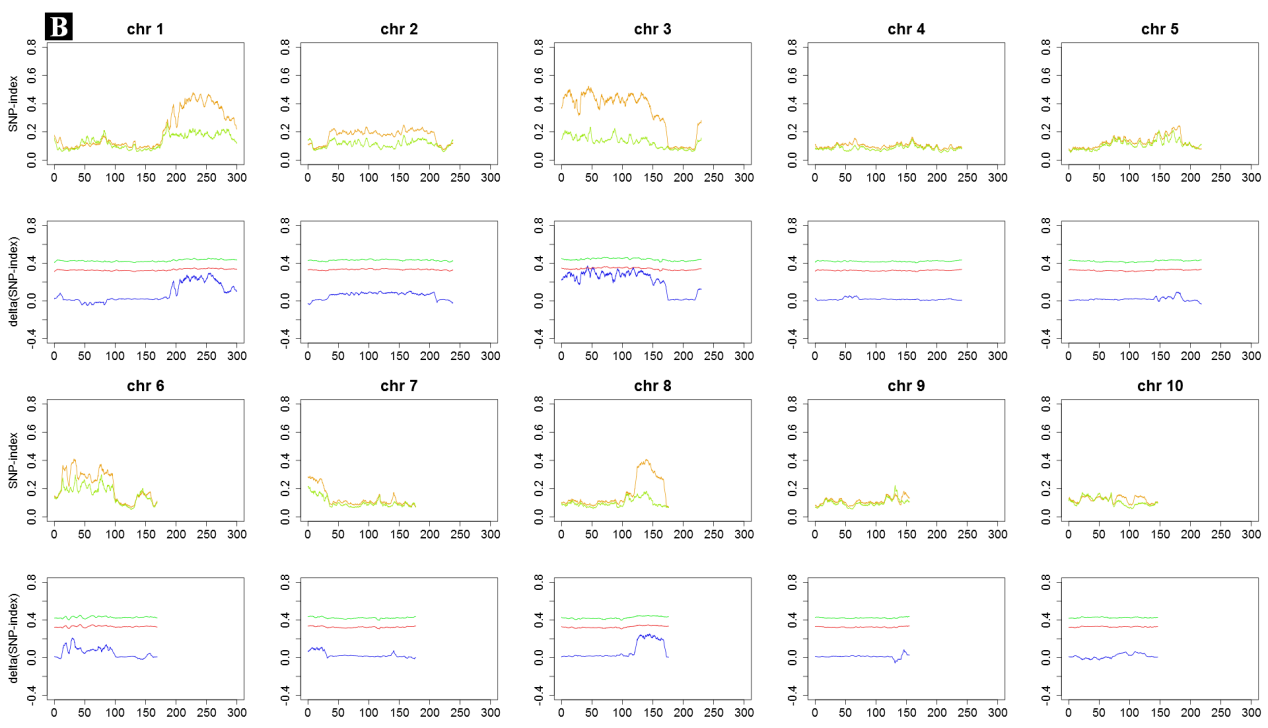


Figure S2. continued


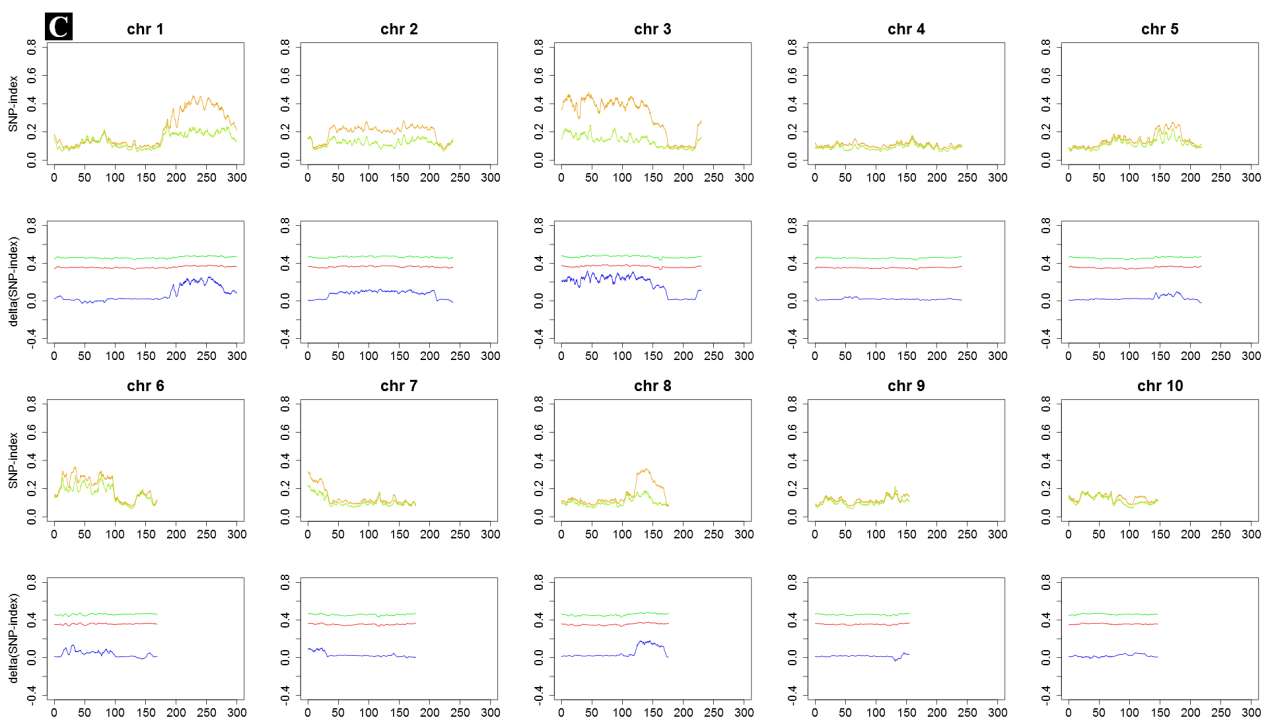

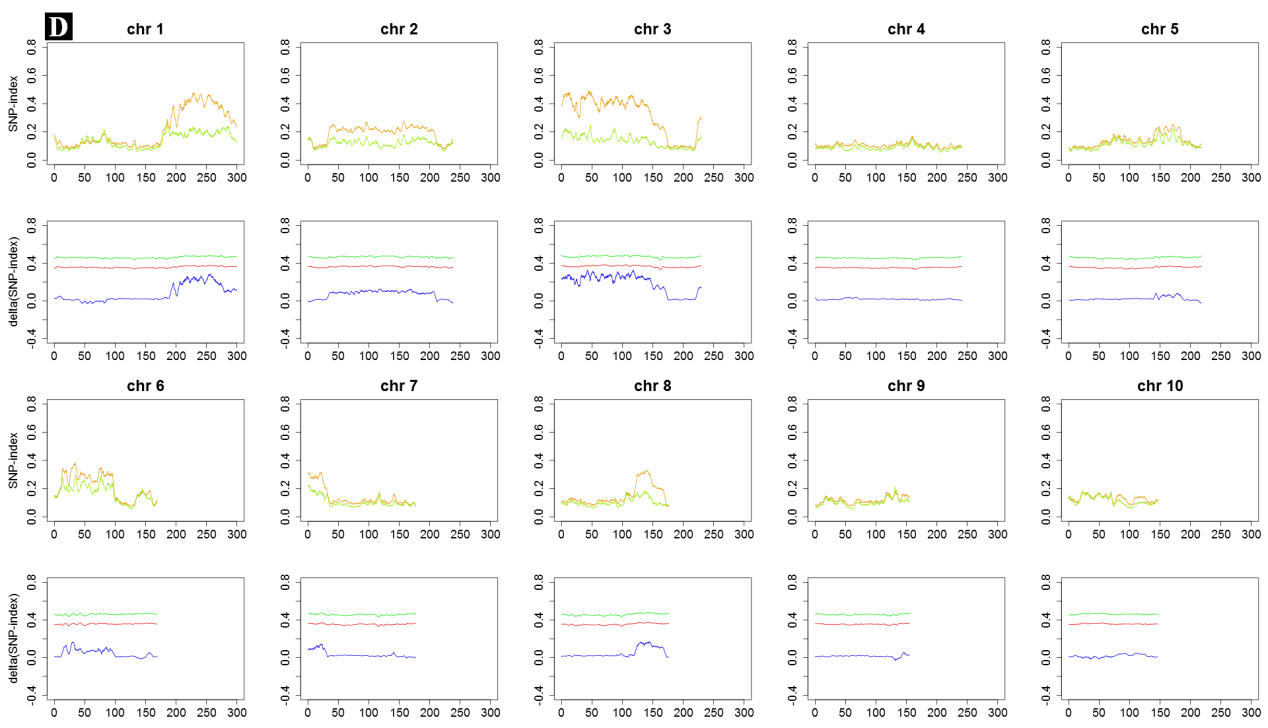


Figure S2. continued


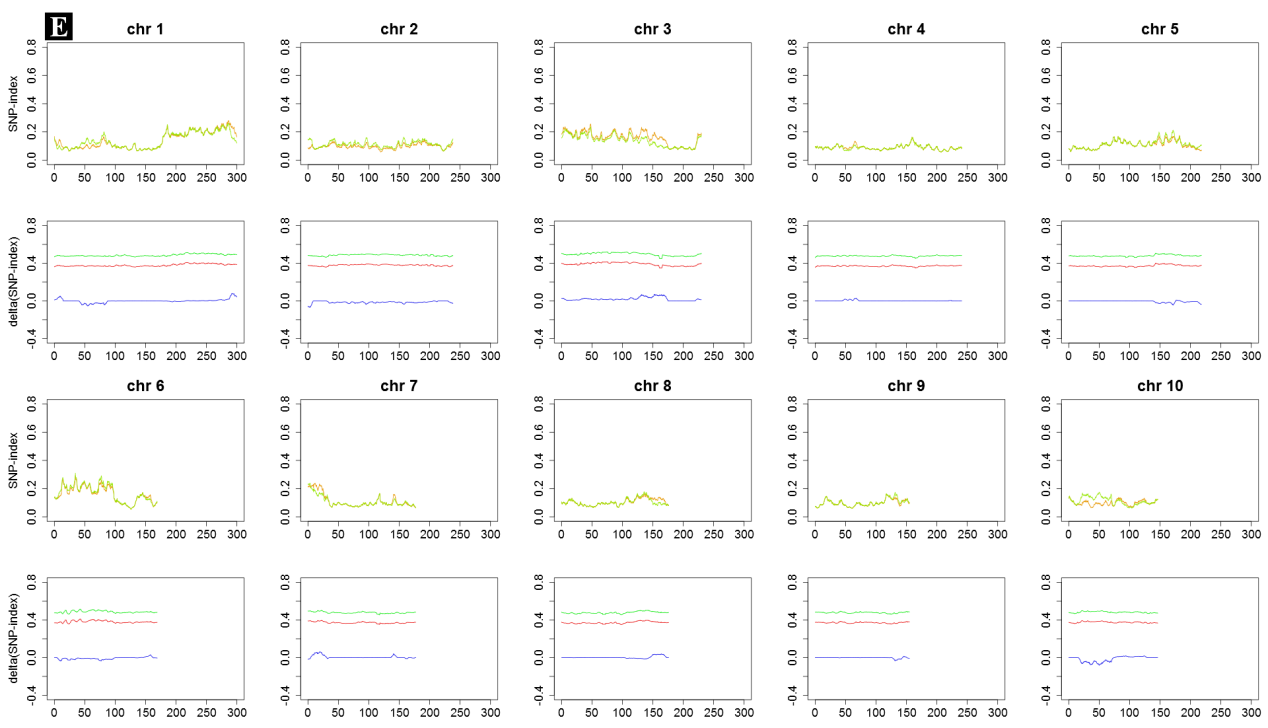


**Figure S2.** SNP-index graphs of high PEDS bulks and low PEDS bulk, and Δ (SNP-index) graphs in three environments. (**A**) 14EEDS environment, (**B-D**) 14JH environment, and (**E**) 15JH environment. X-axis represents the position of chromosome (Mb) and Y-axis represents the SNP-index or Δ (SNP-index). The light green lines represent the SNP-index of low PEDS bulks, the orange lines represent the SNP-index of high PEDS bulks, the blue lines represent the Δ (SNP-index), the red lines represent the threshold values under the null hypothesis at significant level P < 0.05, and the green lines represent the threshold values under the null hypothesis at significant level P < 0.01.


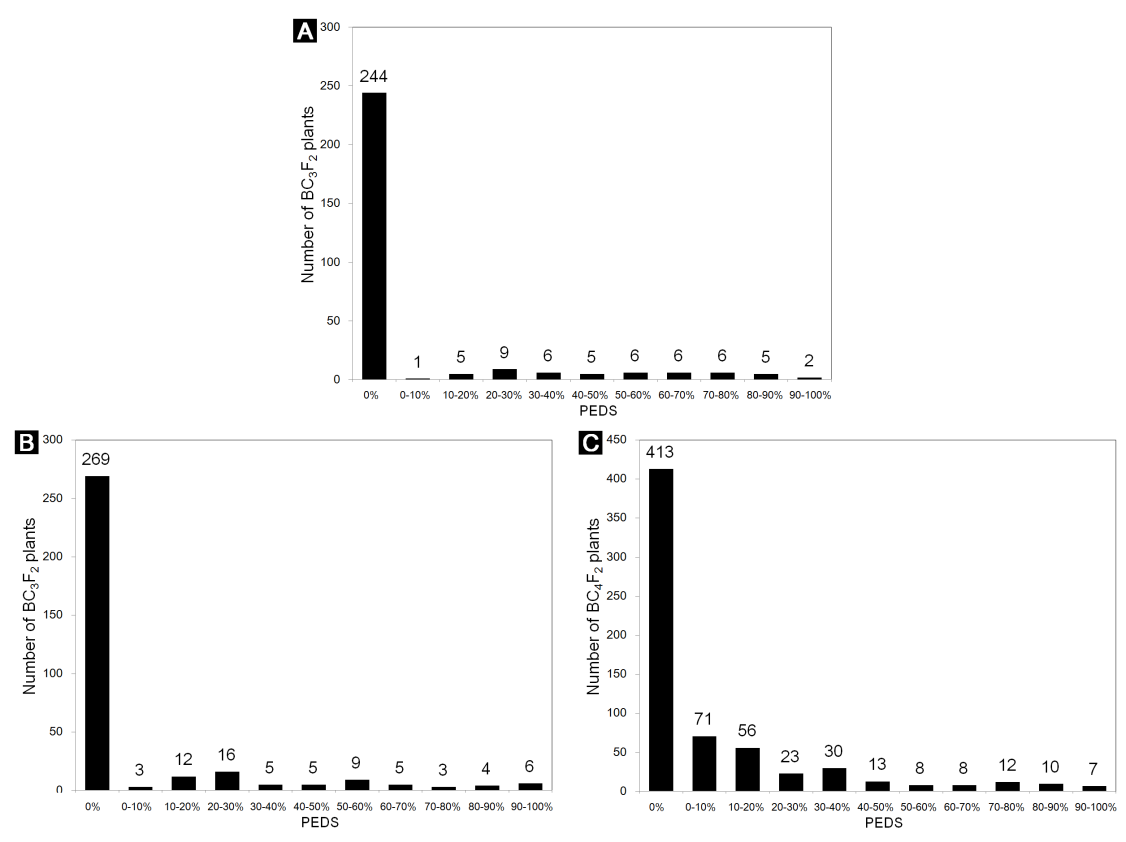


**Figure S3.** Frequency distribution of BC_3_F_2_ and BC_4_F_2_ populations used for traditional QTL mapping in three environments. (**A**) The BC_3_F_2_ population in the 14JH environment, (**B**) The BC_3_F_2_ population in the 18WJ environment, and (**C**) The BC_4_F_2_ population in the 15WJ environment.
