# Supplementary material for: Combining QTL-seq and linkage mapping to uncover the genetic basis of single vs. paired spikelets in the advanced populations of two-ranked maize×teosinte
Source: BMC Plant Biol. 2021 Dec 4;21:572. doi: 10.1186/s12870-021-03353-3 (PMC8642974; doi:10.1186/s12870-021-03353-3)
Supplement: Supplementary file 2 — Additional file 2: Table S1. Sequencing of maize parent line and bulks, and mapping of sequence reads. Table S2. The information on markers used in traditional QTL mapping. [file 12870_2021_3353_MOESM2_ESM.docx]

**Table S1** Sequencing of maize parent line and bulks, and mapping of sequence reads

| Sample ^a^ | Phenotype | Number of plants | Number of reads (M) | Bases (G) | Q20 (%) | Genome coverage (%) | Average depth (×) |
| --- | --- | --- | --- | --- | --- | --- | --- |
| SICAU12122 | PEDS=0% | 5 | 117.17 | 34.98 | 97.91 | 80.24 | 15.21 |
| HP1-bulk | 90%<PEDS≤100% | 50 | 293.66 | 44.05 | 95.48 | 82.34 | 19.15 |
| HP2-bulk | 90%<PEDS≤100% | 50 | 293.43 | 44.02 | 94.88 | 83.02 | 19.16 |
| HP3-bulk | 90%<PEDS≤100% | 50 | 293.91 | 44.09 | 93.91 | 85.20 | 19.16 |
| HP4-bulk | 90%<PEDS≤100% | 50 | 293.47 | 44.02 | 95.85 | 85.12 | 19.14 |
| HP5-bulk | 0%<PEDS≤10% | 50 | 293.92 | 44.09 | 94.03 | 81.43 | 19.17 |
| LP-bulk^b^ | PEDS=0% | 50 | 294.18 | 44.13 | 93.67 | 83.34 | 19.18 |

^a^ Short reads were aligned to the maize reference genome (B73-RefGen_v4) that downloaded from MaizeGDB (*http://www.maizegdb.org/*).

**Table S2** The information on markers used in the traditional QTL mapping

| Marker | Chromosome | Physical position (Mb)^a^ | Forward sequence (5'-3') | Reverse sequence (5'-3') |
| --- | --- | --- | --- | --- |
| PM26 | 1 | 178.92 | AGAGACGTACGGGAAGACGA | ACTCACTGGTTGCTGTCACG |
| PM1 | 1 | 184.63 | ACGATCAGTGAGTGCGGTTA | TGCCTTGTTATTGTAGCCGA |
| PM2 | 1 | 222.02 | ATCGTCATCAGCGAATGGA | TGACCTCTGGACACCAGTTG |
| PM3 | 1 | 230.13 | ACTAAATAAATGCCACACGA | TAGTACTGCCTAACACATGC |
| PM4 | 1 | 258.06 | CTATAACAGAACAACTTGCG | CCTATTCAATAGCTTCCCTA |
| PM5 | 1 | 279.92 | GACCCCAAATCTCTCCTTCCTC | TAGCTAAGCTTGTGCTTGCTCG |
| PM27 | 1 | 281.12 | CATTGTCGGAGCGGACTC | CTAAACCCATTGTCGTCGGA |
| PM28 | 1 | 292.90 | TGAGATTGTAGGGAAAGACCAGA | TGAAATGCCAAATCCCAAAG |
| PM29 | 3 | 2.93 | GAATGAATGGGTTGGTCGTG | GGACGCCACCACTCTAGTCT |
| PM6 | 3 | 9.17 | GGCGAATACGATGCAAGATA | CCTCATCTCCTGCCACTCTT |
| PM7 | 3 | 10.86 | GAATTCTAGGTGTAGCTTCGGGT | TAGAATTTTCGCAAACATCGCA |
| PM30 | 3 | 11.89 | GACCAGAAGTGAGCTAAAGTGG | GCAAAAGAAGATGACGTGTGA |
| PM8 | 3 | 12.56 | CTTTTGCATGAGCTACCCG | TTTCATTCTATTGTTCGGTTGC |
| PM9 | 3 | 12.83 | AAAAATCCCATTCAAATAAC | CAGGCTCCTCCTTTTTCTCTG |
| PM10 | 3 | 14.12 | GCTTTTGTTATTTTCATTCGATCA | ATCCAAACAAATAAAAGAACC |
| PM11 | 3 | 39.65 | TCTAGGGAGAGAACCCGTCA | AGAAACGATGTGATGCATGG |
| PM12 | 3 | 50.46 | ACACGGTCCCGGTACATCT | GTGCGACAAGTGTTGCGTC |
| PM13 | 3 | 67.93 | GCTGCCAGCCTAACAAATTC | GCATTGCAAGGTCTTTCTCC |
| PM14 | 3 | 128.62 | CTTGCATTGTGCTGTTTAGGA | GAGAACAGCAACAGAAGGACA |
| PM15 | 3 | 140.62 | TTCGGGGTTTAGGACACTTG | AGGTAGGCGAATTGCATGAG |
| PM32 | 3 | 153.35 | ACTCAACCACTCACTCACCC | CATGCAGAGGTAGCACTAGC |
| PM16 | 3 | 157.37 | CCGTGAAACGCTTGTGATT | GCGGATGAAGAAAAGTCACC |
| PM17 | 6 | 21.26 | GTCAATTGCGGTCGCGTA | TAGTCGCGATCAGTCACACC |
| PM18 | 6 | 75.43 | TGGTGATCATCAGTTCCCGT | CTCGTCCCTGTCGTAGAACG |
| PM19 | 6 | 83.98 | GTGGAAGGGGAGGTCGTT | TCCCCAGAGTAGTGTGGTTG |
| PM20 | 6 | 96.57 | GTTTGAGGAGCGTGCGAC | ATCCATTGTTCCCCCTTGAG |
| PM21 | 8 | 111.75 | GTGACAGGAGAAGAGGCACG | CAACTGTCGTCCGACCTTC |
| PM22 | 8 | 116.80 | TTTTCTATTCTCGCTGCCGT | GGCTCCTTTAGAGAAACCTTCA |
| PM23 | 8 | 126.24 | CCTCCCTGCATCCTGCTA | TGCGGAACCATATGGAATTT |
| PM24 | 8 | 135.14 | GGAGCATCAACAAGCTTTCC | GAGGAGCTACGGGTGTGGT |
| PM25 | 8 | 149.80 | GACGAGCCGAGAGCAAAAC | CGCACCATCTAGCCATCAG |

^a^ Physical position is based on B73-RefGen_V4 sequence
